# Endocrine dysfunction following traumatic brain injury: a 5-year follow-up nationwide-based study

**DOI:** 10.1038/srep32987

**Published:** 2016-09-09

**Authors:** Wei-Hsun Yang, Pau-Chung Chen, Ting-Chung Wang, Ting-Yu Kuo, Chun-Yu Cheng, Yao-Hsu Yang

**Affiliations:** 1 Division of Neurosurgery, Department of Surgery, Chang Gung Memorial Hospital, Chia-Yi Center, Taiwan; 2 Institute of Occupational Medicine and Industrial Hygiene, National Taiwan University College of Public Health, Taipei, Taiwan; 3 Department of Environmental and Occupational Medicine, National Taiwan University Hospital and National Taiwan University College of Medicine, Taipei, Taiwan; 4 Center of Excellence for Chang Gung Research Datalink, Chang Gung Memorial Hospital, Chiayi, Taiwan; 5 Department of Traditional Chinese Medicine, Chang Gung Memorial Hospital, Chia-Yi, Taiwan; 6 School of Traditional Chinese Medicine, College of Medicine, Chang Gung University, Taoyuan, Taiwan

## Abstract

Post-traumatic endocrine dysfunction is a complication of traumatic brain injury (TBI). However, there is lack of long-term follow-up and large sample size studies. This study included patients suffering from TBI registered in the Health Insurance Database. Endocrine disorders were identified using the ICD codes: 244 (acquired hypothyroidism), 253 (pituitary dysfunction), 255 (disorders of the adrenal glands), 258 (polyglandular dysfunction), and 259 (other endocrine disorders) with at least three outpatient visits within 1 year or one admission diagnosis. Overall, 156,945 insured subjects were included in the final analysis. The 1- and 5-year incidence rates of post-traumatic endocrinopathies were 0.4% and 2%, respectively. The risks of developing a common endocrinopathy (p < 0.001) or pituitary dysfunction (P < 0.001) were significantly higher in patients with a TBI history. Patients with a skull bone fracture had a higher risk of developing pituitary dysfunction at the 1-year follow up (p value < 0.001). At the 5-year follow up, the association between intracranial hemorrhage and pituitary dysfunction (p value: 0.002) was significant. The risk of developing endocrine dysfunction after TBI increased during the entire 5-year follow-up period. Skull bone fracture and intracranial hemorrhage may be associated with short and long-term post-traumatic pituitary dysfunction, respectively.

Traumatic brain injury (TBI) is a common cause of disability and death among adults worldwide. The incidence is from 91/100,000 in Spain^1^ to 300/100,000 in Italy^2^. In the U.S., 230,000 patients are admitted with a head injury annually^3^. These figures demonstrate admitted cases; therefore, the actual number could be higher. Survivors of TBI face a variety of complications in the future, such as impaired movement, seizure, or hydrocephalus. Post-traumatic endocrinopathies have been reported among these complications. This phenomenon was first reported in 1918^4^ and was originally thought to be a rare complication^5^. Subsequently, more and more studies reported a wide range of incidence rates from 2% to 90%^6^,^7^,^8^,^9^. An endocrinopathy can cause serious physical and mental effects in patients with TBI^10^,^11^,^12^. Therefore, the quality of life in these patients could be severely impaired. For example, depression and fatigue caused by hypopituitarism^13^, neuropsychiatric issues caused by thyroid hormone disorders^10^, electrolyte imbalance^14^, diabetes insipidus^15^, decreased cardiac function, and increased cardiovascular disease due to growth hormone deficiency^16^,^17^ are some of the possible effects impairing quality of life.

Numerous studies have described the risk of developing a post-traumatic endocrinopathy^14^,^18^,^19^,^20^. Several articles also suggest the need to screen patients with a TBI history at 3 and 12 months^21^,^22^,^23^,^24^, even if they present with nonspecific symptoms, such as fatigue, impaired concentration, or depression. However, several review articles have reported a number of limitations on the part of previous studies on post-traumatic endocrinopathies, including small sample size^25^ and choice of diagnostic criteria^26^. Besides, the risk and association has not been clarified^27^.

The other limitation in this respect is the lack of long-term follow up. Some studies have mentioned possible resolution of pituitary dysfunction 1 year after TBI^28^,^29^,^30^. Aimaretti *et al*. reported possible improvement or worsening over time^23^,^31^. Krahulik *et al*. reported recovery of hormonal function after 6 months^32^. Agha *et al*. described a patient who spontaneously recovered from hypopituitarism after 5 years^33^. Therefore, long-term follow up has been recommended by many review articles^14^,^27^. However, most studies have a median 1-year follow up, and only a few studies or a few patients had a follow-up time longer than 1 year^29^,^34^,^35^. Therefore, larger populations and longer follow-up periods are needed to confirm the association.

The National Health Insurance Research Database (NHIRD) was established by the National Health Research Institutes of Taiwan, and includes all medical claims data from 26 million enrollees from 1996 to 2009. This database covers >98% of the Taiwanese population over a period of 14 years. Due to the large population and long-term follow up period, this study used unique NHIRD Taiwanese data to explore the long-term risk of developing post-traumatic pituitary dysfunction in patients with TBI.

## Materials and Methods

### Data source

The Taiwanese government implemented the National Health Insurance program in March, 1995; this program provides general health insurance coverage to almost the entire Taiwanese population. The National Health Insurance Research Database (NHIRD) for this program contains the registration files and original reimbursement claims data maintained by the National Health Research Institutes (NHRI). The NHRI has provided these data to scientists for research purposes since 2000. The NHIRD contains medical information, including data on medical care facilities and specialties, information on prescriptions, including the names of prescribed drugs, dosages, prescription duration, and total expenditures, operations and examinations, patient sex and birth date, date of visit or hospitalization, transfer identification number, and diagnoses coded in the International Classification of Diseases, 9th Revision, Clinical Modification (ICD-9-CM) format. The NHRI extracted one million randomly sampled representative data from the registry of all enrollees and created the Longitudinal Health Insurance Database in 2005 (LHID 2005), which is representative of all beneficiaries.

This study adhered to strict confidentiality guidelines, in accordance with regulations regarding personal electronic data protection, and was approved by the ethics review board of the Chang Gung Memorial Hospital, Chia-Yi Branch(No: 103-0504B). The data were analyzed anonymously and the requirement for informed consent was waived by institution of review board.

### Study subjects and design

**Figure 1:**
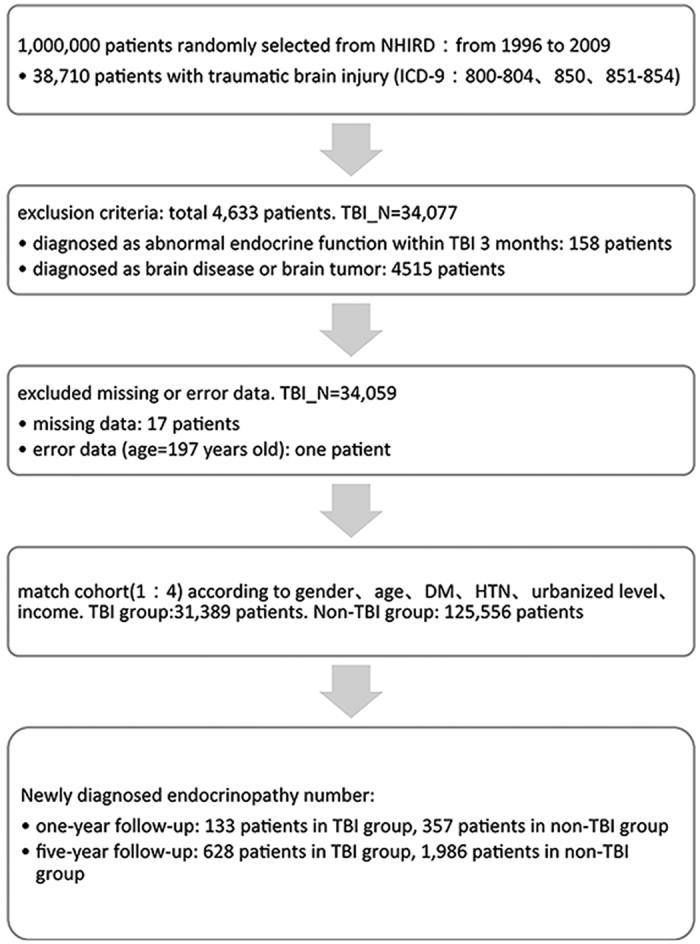
Flow diagram of this nationwide-based study.

The flow diagram of this nationwide-based study is shown in Fig. 1. This study included patients who suffered from TBI (ICD9:800–804, 850–854) during 1996–2009. All medical records of the TBI cohort were extracted and analyzed, and all enrolled study subjects were followed up until death or the end of 2009. Endocrine disorders were identified using the following ICD codes: 244 (acquired hypothyroidism), 253 (pituitary dysfunction), 255 (adrenal gland disorders), 258 (polyglandular dysfunction), and 259 (other endocrine disorders) with at least three records of outpatient visits within 1 year or one admission diagnosis during the study period.

We excluded subjects with endocrine dysfunction, stroke (ICD9:430–438), or brain tumor (ICD9: 191, 225.0, 225.1, 225.2) diagnosed before the TBI event. Subjects with data errors or missing data were also excluded. The TBI subjects and non-TBI subjects were frequency matched randomly by age, sex, income, urbanization, diabetes, and hypertension at a ratio of 1:4 (TBI subjects vs. non-TBI user). Overall, 156,945 insured subjects (31,389 matched sets) were included in the final analysis (Fig. 1).

### Statistical analysis

We used the Kaplan–Meier method to calculate survival curves and the log-rank test to detect differences in the survival curves. Finally, Cox proportional hazards models were used to compute the hazard ratios (HRs) and 95% confidence intervals (CIs) after adjustment for comorbidities and sociodemographic characteristics (age, sex, income, and level of urbanization). Urbanization levels in Taiwan are divided into four strata according to a Taiwan National Health Research Institute publication. Level 1 refers to the most urbanized community and level 4 refers to the least urbanized community. All analyses were conducted using SAS ver. 9.4 software (SAS Institute, Cary, NC, USA).

## Results

### Post-traumatic endocrinopathies

The demographic characteristics and selected morbidities are shown in Table 1. There were 31,389 patients in the TBI group and 125, 556 patients in the non-TBI group. Mean age and the sex distribution were similar because the non-TBI group was age- and sex-matched with the original cohort. In addition, we also decreased the effect of covariates, such as diabetes mellitus, hypertension, and heart disease; therefore, the proportions of these covariates are similar in these groups. Among them, 133 (0.4%) patients in the TBI group and 357 (0.3%) patients in the non-TBI group were newly diagnosed with an endocrine disorder at the 1-year follow up. The incidence of endocrine dysfunction was significantly higher in the TBI group than that in the non-TBI group (p value < 0.0001).

Patients in the TBI group seemed to have a higher risk of developing an endocrine disorder at the 1-year follow up compared with the non-TBI group, after adjusting for age, sex, and comorbidities (adjusted HR: 1.49; p < 0.001; Table 2).

### Pituitary dysfunction

We suspected a higher risk of developing pituitary dysfunction, as opposed to another endocrinopathy, in patients with TBI because the pituitary gland is located intracranially. Therefore, we separated all endocrinopathies into two groups of pituitary and non-pituitary dysfunction according to their ICD-9 code.

Thirty-five (0.1%) patients in the TBI group and 80 (0.1%) patients in the non-TBI group were newly diagnosed with a pituitary disorder during the 1-year follow-up period (Table 1). Using multivariable regression analysis, patients in the TBI group demonstrated a significantly higher risk of developing a pituitary disorder during the 1-year follow up when compared with the non-TBI patients (adjusted HR: 2.06; p < 0.001; Table 3).

### Types of TBI

Previous studies have reported an association between different types of brain injury and the risk of developing a post-traumatic endocrinopathy. We separated the patients with TBI into mild head injury (ICD-9: 850), head injury with intracranial hemorrhage (ICD-9: 851–854), and skull bone fracture (ICD-9: 800–804). Table 1 shows that 11,063 (35.2%) patients had mild head injury, 14,940 (47.6%) had head injury and intracranial hemorrhage, and 5,386 (17.2%) had a skull bone fracture.

The association between mild head injury and the risk of developing an endocrinopathy was not significant in the multivariate analysis (p value: 0.064, HR: 1.34; Table 2), but the association is significant in the group with hemorrhagic head injury (p value: 0.007, HR: 1.44). Moreover, the association was more significant in the skull bone fracture group (p value < 0.001, HR: 2.05).

The association between mild head injury and a pituitary disorder remained insignificant (p value: 0.065, HR: 1.78; Table 3). In addition, the association between intracranial hemorrhage and pituitary dysfunction was not significant. However, the association was well demonstrated in the skull bone fracture group of patients (p value < 0.001, HR: 3.77).

### Five-year follow-up

**Figure 2:**
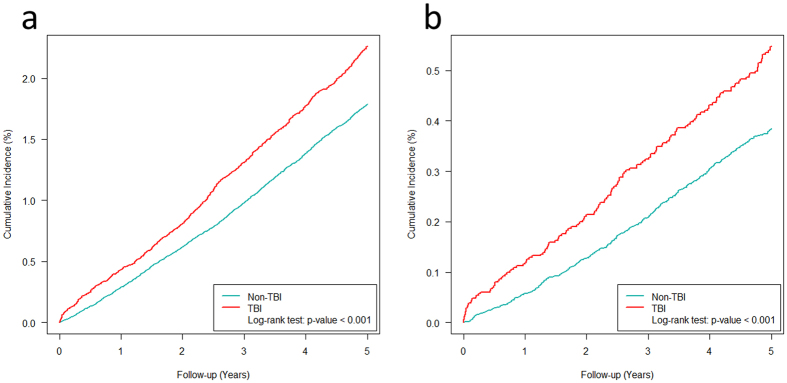
(**a**) Cumulative incidence curves for post-traumatic common endocrinopathies during the 5-year follow-up period. (**b**) Cumulative incidence curves for post-traumatic pituitary dysfunction during the 5-year follow-up period.

According to Fig. 2a,b, the incidence of post-traumatic endocrinopathy and pituitary dysfunction increased linearly over the 5-year period, and the gap between patients with and without TBI increased gradually. Because previous studies have described possible resolution or worsening of an endocrinopathy during a long-term follow up, we extended our follow up to 5 years to explore whether there was any change in the association between TBI and endocrinopathies.

The association between post-traumatic endocrinopathies and TBI remained at the 5-year follow up (p value < 0.001, HR: 1.27; Table 2). However, the association between a pituitary disorder and skull bone fracture was not significant at 5 years (p value 0.134, HR: 1.41; Table 3), but the association between pituitary dysfunction and intracranial hemorrhage became significant (p value: 0.002, HR: 1.46).

## Discussion

This is the first study to describe long-term follow-up results of the incidence of post-traumatic endocrinopathies using a nationwide population. We noted that the incidence of endocrinopathies and pituitary dysfunction increased gradually during the 5-year follow up after TBI. Patients with traumatic brain injury had a higher risk of developing an endocrinopathy (p value: < 0.0001) and pituitary dysfunction (HR: 2.06 at 1-year, HR: 1.27 at 5-year) at the 1- and 5-year follow-ups. We also explored the different effects among the three types of brain injury. Among the three types, a skull bone fracture had the most significant association with pituitary dysfunction during the 1-year follow-up period. However, intracranial hemorrhage had a stronger association with pituitary dysfunction than that of skull bone fracture during the 5-year follow-up period.

Post-traumatic endocrinopathy was first reported in 1918^4^, and many studies have reported follow ups for decades. However, incidence has varied from 5% to 90%^36^. A lower incidence (5.4%) was reported by Kokshoorn^8^ *et al*. A systematic review article by Lauzier *et al*. reported that about one third of TBI cases developed later pituitary dysfunction^25^ The incidence of pituitary injury at autopsy is 26–86%^37^,^38^,^39^.

In our study, the incidence of post-traumatic endocrinopathies at the 1-year follow up was 0.4%, which is far lower than most studies. Some of the reasons for this low incidence rate are, initially, that clinicians tend to attribute non-specific symptoms such as headache, dizziness, and extremity weakness to head injury sequelae rather than to an endocrinopathy. Consequently, patients seldom receive a hormonal evaluation to confirm the diagnosis.

Second, patients with severe head injury usually present with lethargy, stupor, or even coma. It is difficult for clinicians to assess subtle changes in consciousness and to investigate the possibility of changes in hormone levels in patients with low coma scale scores. In addition, we were unable to obtain clinical data, such as the coma scale score and duration of the ICU stay, from the NHIRD. The combined lack of severity data and assessments of hormonal changes in patients with extremely severe head injury likely contributed to the low incidence of post-traumatic endocrinopathy identified in our study.

Third, selecting a reliable test is difficult, particularly when the patient is in the intensive care unit^31^. A variety of tests have been used to assess hormone levels, and there is a wide range of diagnostic cut-off values^40^,^41^,^42^,^43^. Additionally, Klose *et al*. have pointed to the unreliability of hormone assessments during the acute phase of brain injury^27^.

Some differences were noted between the populations with and without TBI. The incidence of endocrinopathies differed between the groups, as patients with TBI had a higher risk of developing an endocrinopathy (p value: 0.0001). However, the ICD-9 codes we used included pituitary dysfunction, hypothyroidism, adrenal gland and cortisol-secreting diroders, and others. Many studies have discussed the effects of TBI on different hormone abnormalities^22^,^23^,^25^. In particular, the pituitary gland is more vulnerable to injury because it is located intracranially.

The differences between the TBI and non-TBI groups still existed when we evaluated pituitary dysfunction. Patients with a TBI history had a higher chance of developing pituitary dysfunction. In contrast, the difference in the risk of developing a non-pituitary abnormality was less significant between the TBI and non-TBI groups.

This result shows that TBI may have a greater effect on pituitary than on other non-pituitary hormone tissues because the pituitary gland is located inside the cranial vault and could face direct impact from the injury, whereas other endocrine glands, located outside the cranial vault, would not have the same level of risk. As a result, a patient with a TBI injury history would have a higher chance of developing pituitary dysfunction compared with a non-pituitary abnormality.

Wachter *et al*. reported that the increased intracranial pressure caused by brain edema is a mechanism for pituitary dysfunction after TBI^44^. Salehi *et al*. also stated that brain injury from a shearing force could result in a hypothalamic-pituitary injury^45^. Hypoxia following brain injury also causes pituitary dysfunction^6^.

Previous studies have reported a variety of effects from different types of brain injuries^27^,^46^,^47^,^48^. Aimaretti *et al*. reported that subarachnoid hemorrhage is a risk factor for pituitary dysfunction^31^. In our study, we subgrouped patients with TBI into three ICD-9 code groups of mild head injury, intracranial hemorrhage, and skull bone fracture.

According to our results, the risk of developing a future endocrinopathy was less significant in patients with only mild head injury, (HR: 1.34); however, if a patient had an intracranial hemorrhage or a skull bone fracture, risk increased. In particular, the HR was 2.05 in the skull bone fracture group. Furthermore, the risk of developing pituitary dysfunction in patients with mild head injury was not as significant (HR: 1.78). Intracranial hemorrhage and skull bone fracture both increased the risk of developing pituitary dysfunction, and the association was the strongest in the skull bone fracture group (HR: 3.77).

Several reasons could explain the strong effect of skull bone fracture on pituitary dysfunction. First, the pituitary gland is situated above the sphenoid sinus where it is in close contact with the surrounding skull bone. The impact causing a head injury and skull bone fracture could be transmitted to the pituitary gland by direct contact. However, if an intracranial hemorrhage is located remotely to the pituitary gland, less injury is likely to be caused. The direct force could cause vessel injury, particularly the long hypophyseal vessel and portal capillaries in the stalk that supply the pituitary gland^39^. Second, impact power differs, as the energy needed to cause a skull bone fracture is probably greater than the energy required to cause a mild head injury. Therefore, a higher impact would result in a greater chance of pituitary injury and later dysfunction.

Furthermore, according to a review by Kokshoorn *et al*., most studies have a median 1-year follow-up time^43^. However, some studies have reported on the long-term effect of TBI^7^,^21^,^29^,^39^. Aimaretti *et al*. reported possible improvement or worsening in pituitary function after a 3-month follow-up^31^. Other studies have reported the possible resolution of pituitary dysfunction after 1 year.

Therefore, patients with brain injury need a long-term follow up. We noted a linear pattern of cumulative incidence of post-traumatic endocrinopathies during 1996–2009, and a gradual widening of the gap between the groups with and without TBI. The incidence of pituitary dysfunction after TBI also showed a similar pattern. This result reveals the possibility of long-term, persistent effects of TBI at the endocrine level.

When we evaluated the risk of pituitary dysfunction among the three types of brain injury at the 5-year follow-up, the association between skull bone fracture and pituitary dysfunction became less significant compared with the association at the 1-year follow up. In contrast, the association between intracranial hemorrhage and pituitary dysfunction became stronger. However, the reasons for these differences are unknown.

There are some possible explanations. First, the impact that causes a skull bone fracture lasts for only a short period, which would only cause short-term pituitary dysfunction. Short-term hormone dysfunction could also be explained by acute phase inflammation and stress^49^,^50^,^51^,^52^.

Second, although the impact causing an intracranial hemorrhage lasts for a short time, once the hemorrhage occurs, it can mean long-term neuron functional loss. Shallice *et al*. reported that disrupting brain networks could cause altered cognition and behavior^53^. Therefore, it is reasonable to consider that the risk of developing pituitary dysfunction increases in patients with long-term cerebral dysfunction.

Some limitations of our study should be mentioned. First, selection bias could not be avoided completely, although patients diagnosed with endocrine dysfunction before the TBI event were excluded. However, patients with a pre-existing endocrinopathy but with subclinical symptoms would probably not have undergone endocrine testing. These patients would not have been recognized as such and therefore would not have been excluded from our study. However, this error would have occurred randomly, since the same definitions/criteria were used for the two groups, likely resulting in a similar incidence of endocrine dysfunction in both groups. Second, the lack of actual laboratory data in the NHIRD databases, the kind of hormone measurements that could be extracted, and some examination results were not recorded. Consequently, we were unable to further assess the types of hormone dysfunction. Third, we could not differentiate TBI severity among patients with intracranial hemorrhage and skull bone fracture. For example, no data are available in the database regarding the coma scale score, pupil size, or muscle power. In addition, imaging information is unavailable. Therefore, we were unable to further explore the association between TBI severity and the risk of developing an endocrinopathy. In addition, details of the TBI mechanism are not recorded in the NHIRD, such as falls and pedestrian-related, motor vehicle-related, sports, and work injuries. As a result, we were unable to analyze the potential relationship between the TBI mechanism and the risk of endocrinopathy. The fourth limitation was that some patients with TBI may not have been recorded in the database because patients with mild head injury do not always seek medical help and would not be admitted to a hospital. This population could further decrease the risk of post-traumatic endocrinopathy and cause an over-estimate in our study.

## Conclusion

In conclusion, we report long-term follow-up results on the incidence of post-traumatic endocrinopathies using nationwide population. Patients with TBI had a higher risk of developing an endocrinopathy and pituitary dysfunction at the 1- and 5-year follow-ups. Among the three brain injuries, skull bone fracture was most significantly associated with pituitary dysfunction during the 1-year follow-up period. However, intracranial hemorrhage had a stronger association with pituitary dysfunction than that of skull bone fracture during the 5-year follow-up period. Long-term monitoring of pituitary function is recommended in patients with a TBI history due to the long-term effects of brain injury on the pituitary.

## Additional Information

**How to cite this article**: Yang, W.-H. *et al*. Endocrine dysfunction following traumatic brain injury: a 5-year follow-up nationwide-based study. *Sci. Rep.* **6**, 32987; doi: 10.1038/srep32987 (2016).

## Figures and Tables

**Table 1:** baseline characteristics of the TBI group and the non-TBI group.

Variables	TBI (N = 31,389)		Non TBI (N = 125,556)		P-value
	count	%	count	%	
Gender					
Male	19,024	60.6	76,096	60.6	NS
Female	12,365	39.4	49,460	39.4	
Age	39.75 ± 19.18	39.75 ± 19.18			
<18	3,272	10.4	13,088	10.4	NS
18–45	16,472	52.5	65,888	52.5	
>45	11,645	37.1	46,580	37.1	
Covariates					
DM					
Yes	2,735	8.7	10,940	8.7	NS
No	28,654	91.3	114,616	91.3	
HTN					
Yes	6,123	19.5	24,492	19.5	NS
No	25,266	80.5	101,064	80.5	
heart disease					
Yes	2,685	8.6	10,682	8.5	NS
No	28,704	91.4	114,874	91.5	
Arrhythmia					
Yes	1,528	4.9	5,650	4.5	0.0053*
No	29,861	95.1	119,906	95.5	
Urbanized level					
1(City)	6,182	19.7	24,728	19.7	NS
2	15,684	50.0	62,736	50.0	
3	6,259	19.9	25,036	19.9	
4(Villages)	3,264	10.4	13,056	10.4	
Income (New Taiwan Dollars)					
income = 0	12,682	40.4	50,728	40.4	NS
1< = income < = 15,840	4,959	15.8	19,836	15.8	
15,841< = income < = 25,000	10,771	34.3	43,084	34.3	
Income > = 25,001	2,977	9.5	11,908	9.5	
Endocrine abnormalities(1-year follow-up)					
No	31,256	99.6	125,199	99.7	<0.0001
Yes	133	0.4	357	0.3	
Pituitary	35	0.1	80	0.1	
Non-pituitary	98	0.3	277	0.2	
TBI(1-year follow-up)					
Mild head injury	11,063	35.2	0	0.0	<0.0001
Intracranial hemorrhage	14,940	47.6	0	0.0	
Skull bone fracture	5,386	17.2	0	0.0	
Endocrine abnormalities(5-year follow-up)					
No	30,761	98.0	123,570	98.4	
Yes	628	2.0	1,986	1.6	
Pituitary	138	0.4	383	0.3	<0.0001
Non-pituitary	490	1.6	1,603	1.3	
TBI(5-year follow-up)					
Mild head injury	11,063	35.2	0	0.0	<0.0001
Intracranial hemorrhage	14,940	47.6	0	0.0	
Skull bone fracture	5,386	17.2	0	0.0	

**Table 2:** Cox’s proportional hazards model: risks for a post-traumatic common endocrinopathy during the 1- and 5-year follow-up periods.

Variables	Multivariate(1 year)			Multivariate(5 years)		
	Hazards ratio	95%CI	P value	Hazards ratio	95%CI	P value
Gender	0.29	0.241–0.356	<0.001	0.29	0.262–0.311	<0.001
Age						
18–45	1.49	1.010–2.196	NS	1.49	1.246–1.770	<0.001
>45	1.13	0.725–1.763	NS	1.15	0.943–1.402	NS
Covariates						
DM	1.30	0.976–1.740	NS	1.43	1.262–1.618	<0.001
HTN	1.32	1.011–1.729	NS	1.29	1.150–1.451	<0.001
heart disease	1.65	1.226–2.216	0.001*	1.43	1.258–1.634	<0.001
Arrhythmia	1.46	1.050–2.035	0.025*	1.52	1.313–1.751	<0.001
Urbanized level						
2	1.01	0.804–1.272	NS	0.92	0.834–1.016	NS
3	0.86	0.645–1.148	NS	0.85	0.755–0.964	0.011*
4	0.91	0.646–1.293	NS	0.86	0.740–0.998	0.046*
Income (New Taiwan Dollars)						
1< = income < = 15,840	1.03	0.774–1.365	NS	1.06	0.943–1.199	NS
15,841< = income < = 25,000	1.03	0.813–1.301	NS	1.03	0.932–1.139	NS
Income > = 25,001	1.35	0.975–1.879	NS	1.10	0.943–1.276	NS
TBI	1.49	1.224–1.822	<0.001	1.27	1.160–1.389	<0.001
Mild head injury	1.34	0.983–1.839	NS	1.27	1.106–1.458	0.001*
Intracranial hemorrhage	1.44	1.104–1.888	0.007*	1.28	1.138–1.440	<0.001
Skull bone fracture	2.05	1.373–3.051	<0.001	1.23	0.981–1.535	NS

**Table 3:** Cox’s proportional hazards model: risks for post-traumatic pituitary dysfunction during the 1- and 5-year follow-up periods.

Variables	Multivariate(1 year)			Multivariate(5 years)		
	Hazards ratio	95%CI	P value	Hazards ratio	95%CI	P value
Gender	0.16	0.099–0.252	<0.001	0.11	0.086–0.135	<0.001
Age						
18–45	1.74	0.816–3.715	NS	1.80	1.292–2.502	0.001*
>45	0.77	0.306–1.925	NS	0.54	0.357–0.822	0.004*
Covariates						
DM	2.41	1.207–4.793	0.013*	2.12	1.517–2.955	<0.001
HTN	0.90	0.454–1.784	NS	0.94	0.685–1.292	NS
heart disease	0.96	0.402–2.225	NS	0.88	0.586–1.321	NS
Arrhythmia	1.38	0.568–3.352	NS	1.75	1.210–2.541	0.003*
Urbanized level						
2	1.21	0.748–1.950	NS	1.12	0.898–1.387	NS
3	0.72	0.370–1.411	NS	1.20	0.926–1.559	NS
4	0.92	0.420–1.991	NS	1.34	0.987–1.826	NS
Income (New Taiwan Dollars)						
1< = income < = 15,840	1.31	0.775–2.228	NS	1.18	0.940–1.489	NS
15,841< = income < = 25,000	0.81	0.484–1.359	NS	0.77	0.620–0.966	0.024*
Income > = 25,001	1.46	0.731–2.896	NS	1.50	1.110–2.037	0.008*
TBI	2.06	1.388–3.067	<0.001	1.43	1.190–1.719	<0.001
Mild head injury	1.78	0.965–3.281	NS	1.41	1.066–1.853	0.016*
Intracranial hemorrhage	1.76	1.007–3.064	0.047*	1.46	1.141–1.854	0.002*
Skull bone fracture	3.77	1.942–7.327	<0.001	1.41	0.900–2.208	NS
